# ﻿Description of two new species of the genus *Pteromalus* Swederus (Hymenoptera, Pteromalidae, Pteromalinae) from Xinjiang, China

**DOI:** 10.3897/zookeys.1234.145429

**Published:** 2025-04-11

**Authors:** Qin Li, Ya-Lin Liu, Guo-Hua Yan, Tong-You Zhang, Hui Xiao, Hong-ying Hu

**Affiliations:** 1 College of Life Science and Technology, Xinjiang University, 666 Shengli Road, Tianshan District, Urumqi, Xinjiang, 830046, China Xinjiang University Urumqi China; 2 Xinjiang Key Laboratory of Biological Resources and Genetic Engineering, 666 Shengli Road, Tianshan District, Urumqi, Xinjiang, 830046, China Xinjiang Key Laboratory of Biological Resources and Genetic Engineering Urumqi China; 3 Key Laboratory of Zoological Systematics and Evolution, Institute of Zoology, Chinese Academy of Sciences, Beijing, 100101, China Chinese Academy of Sciences Beijing China

**Keywords:** Key, morphological characters, new taxa, *Orchestessteppensis*

## Abstract

Two new species of *Pteromalus* Swederus (Hymenoptera, Pteromalidae, Pteromalinae), *Pteromalussteppensis* Li & Hu, **sp. nov.** and *Pteromalusxiaomoheensis* Yan & Li, **sp. nov.**, are described and illustrated for the first time. *Pteromalussteppensis* Li & Hu, **sp. nov.** was reared as a primary, solitary ectoparasitoid of the larval and pupal stages of *Orchestessteppensis* Korotyaev, 2016 (Coleoptera, Curculionidae). Figures of its development and its host damage are also provided.

## ﻿Introduction

The genus *Pteromalus* Swederus, 1795 (type species *Ichneumonpuparum* (Linnaeus, 1795)) belongs to the family Pteromalidae, subfamily Pteromalinae, and is distributed in the all the zoogeographical regions of the world (UCD 2024). This genus includes about 500 described species, with 375 in Europe alone (Gibson et al. 2024). It is the most speciose genus of the family Pteromalidae and has been taxonomically studied since the 19^th^ century (Maletti et al. 2021). The genus can be recognized by the following combination of characters (Graham 1969; Bouček and Rasplus 1991; Bouček and Heydon 1997; Baur 2015): clypeus striate, its anterior margin truncate or weakly to strongly emarginate, always without a median tooth; flagellum with two anelli and six funicular segments; clava in females symmetrical; prepectus with relatively small upper triangular area; paraspiracular sulci rather deep and usually with some transverse costulae. Most taxonomic or faunistic studies of *Pteromalus* concern the Palaearctic fauna (Graham 1969, 1984; Bouček and Rasplus 1991; Dzhaokmen 1998, 2001; Gijswijt 1999; Mitroiu 2008; Baur 2015; Klimmek and Baur 2018; Maletti et al. 2021; Haas et al. 2021; Yan et al. 2023).

All species of *Pteromalus* with known biology are parasitoids of larvae and pupae of various holometabolous insects, such as Lepidoptera, Coleoptera, gall-forming Hymenoptera (Cynipidae, Tenthredinidae), and Diptera (Tephritidae) (Mitroiu 2008; Baur 2015). They play a crucial role in nature as regulating agents of these phytophagous insects (Bouček and Rasplus 1991; Ishizaki and Ishikawa 2010; Mahdavi and Madjdzadeh 2013; Li et al. 2018; Mbata and Warsi 2019; Haas et al. 2021). Despite their ecological and economic importance, *Pteromalus* species are often difficult to identify because of their diversity and sometimes subtle morphological characters that are used to separate species (Burks et al. 2022). Until now, the genus *Pteromalus* comprised 494 valid species, with only 19 species being recorded from China, including *P.albipennis* Walker, 1835; *P.astragali* (Liao, 1987); *P.bifoveolatus* Förster, 1861; *P.chrysos* Walker, 1836; *P.coleophorae* Yang & Yao, 2015; *P.elevatus* (Walker, 1834); *P.miyunensis* Yao & Yang, 2008; *P.orgyiae* Yang & Yao, 2015; *P.procerus* Graham, 1969; *P.puparum* (Linnaeus, 1758); *P.qinghaiensis* Liao, 1987; *P.sanjiangyuanicus* Yang, 2020; *P.semotus* (Walker, 1834); *P.sequester* Walker, 1835; *P.shanxiensis* Huang, 1987; *P.smaragdus* Graham, 1969; *P.temporalis* (Graham, 1969); *P.varians* (Spinola, 1808); and *P.xizangensis* (Liao, 1982) (Liao 1982; Sheng 1985; Liao et al. 1987; Huang et al. 1987; Huang et al. 2003; Yao and Yang 2008; Ye et al. 2012; Yang et al. 2015; Li et al. 2018; Yang et al. 2020; Yan et al. 2023).

The Chinese *Pteromalus* fauna has been poorly studied until now, and many new species, as well as newly recorded species, may be found in China. During a biodiversity expedition of the pteromalid wasps in northern Xinjiang, China, between 2014 and 2022 (funded by the first author’s projects), most *Pteromalus* individuals belonged to species not previously known from Xinjiang. The aim of this work is to review the genus *Pteromalus* from China based on this collected material and data from the literature data (1982–2023), to describe two new species, and to provide the key to the two new species and their similar species.

## ﻿Materials and methods

### ﻿Collected material

All specimens of *Pteromalusxiaomoheensis* Yan & Li, sp. nov. and partial specimens of *Pteromalussteppensis* Li & Hu, sp. nov. were collected with a sweeping net from Xinjiang, China during 2020–2022. Some specimens of *Pteromalussteppensis* Li & Hu, sp. nov. were reared from their hosts during 2014–2016, the rearing process of *Pteromalussteppensis* Li & Hu, sp. nov. and *P.varians* (Spinola, 1808) follows Li et al. (2018). All specimens were mounted, labeled, and examined under a Nikon SMZ 745T stereomicroscope. Images except for *P.tripolii* Graham were taken with a Nikon DS-Fi3 connected to a Nikon SMZ 25 stereomicroscope and the images of *P.tripolii* were downloaded from the interactive key http://pteromalus.identificationkey.fr/mkey.html in Klimmek and Baur (2018). All images were stacked with NIS-Elements software and arranged in figures using Adobe Photoshop. All specimens from Xinjiang, China, including the types of the new species are deposited in the Insect Collection of the College of Life Science and Technology, Urumqi, Xinjiang, China (**ICXU**), and all specimens from the other provinces in China are deposited in the Institute of Zoology, Chinese Academy of Sciences, Beijing, China (**IZCAS**).

### ﻿Morphological description

Morphological terms follow Bouček (1988), Gibson et al. (1997), and Burks et al. (2022). Body length excludes the protruding parts of ovipositor sheaths and was measured in millimeters (mm); other measurements are given as ratios. Abbreviations of morphological terms used are: Fu_n_ = antennal funicular 1, 2…; Gt_n_ = gastral tergite 1, 2…; OOL = shortest distance between eye margin and a posterior ocellus; POL = shortest distance between posterior ocelli.

## ﻿Results

### ﻿Taxonomy

Twenty-one species of *Pteromalus* from China are summarized in Table 1, including 19 species previously reported from China. Among them, there are six species from Xinjiang, including four species reported by Li et al. (2018) (*P.varians* (Spinola, 1808)) and Yan et al. (2023) (*P.elevatus* (Walker, 1834), *P.albipennis* Walker, 1835, *P.temporalis* (Graham, 1969)) and the two new species, *P.steppensis* Li & Hu, sp. nov. and *P.xiaomoheensis* Yan & Li, sp. nov.

**Table 1.** Twenty-one Chinese species of *Pteromalus* and their citation in the Chinese literature between 1982 and 2023.

Num.	Species	Detailed (re)description in Chinese	Distribution in China	Citation	Deposition of type material
1	*P.albipennis* Walker, 1835	Yes	Xinjiang	Yan et al. 2023	**ICXU**
2	*P.astragali* (Liao, 1987)	Yes	Beijing	Liao et al. 1987	**IZCAS**
3	*P.bifoveolatus* Förster, 1861	Yes	Jiangxi, Shandong	Sheng 1985; Huang et al. 1987; Yao and Yang 2008	**IZCAS**
4	*P.chrysos* Walker, 1836	Yes	Fujian	Huang et al. 2003	**IZCAS**
5	*P.coleophorae* Yang & Yao, 2015	Yes	Heilongjiang	Yang et al. 2015	**IZCAS**
6	*P.elevatus* (Walker, 1834)	Yes	Xinjiang	Yan et al. 2023	**ICXU**
7	*P.miyunensis* Yao & Yang, 2008	Yes	Beijing	Yao and Yang 2008	**IZCAS**
8	*P.orgyiae* Yang & Yao, 2015	Yes	Ningxia	Yang et al. 2015	**IZCAS**
9	*P.procetus* Graham, 1969	Yes	Beijing	Yao and Yang 2008	**IZCAS**
10	*P.puparum* (Linnaeus, 1758)	No	Jiangsu, Xinjiang, Xizang, Sichuan, Yunnan, Zhejiang	Liao 1982; Liao et al. 1987	**ICXU, IZCAS**; new record in Xinjiang
11	*P.qinghaiensis* Liao, 1987	Yes	Qinghai	Liao et al. 1987	**IZCAS**
12	*P.sanjiangyuanicus* Yang, 2020	Yes	Qinghai	Yang et al. 2020	**IZCAS**
13	*P.semotus* (Walker, 1834)	Yes	Beijing, Fujian, Jilin, Xinjiang	Huang et al. 1987; Huang et al. 2003; Yao and Yang 2008	**ICXU, IZCAS**; new record in Xinjiang
14	*P.sequester* Walker, 1835	Yes	Inner Mongolia	Ye et al. 2012	**IZCAS**
15	*P.shanxiensis* Huang, 1987	Yes	Shanxi	Huang et al. 1987	**IZCAS**
16	*P.smaragdus* Graham, 1969	Yes	Fujian, Xinjiang	Huang et al. 2003	**ICXU, IZCAS**; new record in Xinjiang
17	*P.steppensis* Li & Hu, **sp. nov.**	Yes	Xinjiang	This study	**ICXU**
18	*P.temporalis* (Graham, 1969)	Yes	Xinjiang	Yan et al. 2023	**ICXU**
19	*P.varians* (Spinola, 1808)	No	Xinjiang	Li et al. 2018	**ICXU**
20	*P.xiaomoheensis* Yan & Li, **sp. nov.**	Yes	Xinjiang	In this study	**ICXU**
21	*P.xizangensis* (Liao, 1982)	Yes	Taiwan, Xizang	Liao 1982	**IZCAS**

The number of teeth on each mandible (both mandibles with four teeth, or left mandible with three teeth and right mandible with four) was not considered a good key character because of the lack of visibility (Gibson et al. 2024), but it is an important differentiating feature for species recognition. Thus, characterize our specimens by reporting the number of mandibular teeth, including the two new species: *P.steppensis* Li & Hu, sp. nov. (both mandibles with four teeth), and *P.xiaomoheensis* Yan & Li, sp. nov. (left mandible with three teeth and right mandible with four). According to Gibson et al. (2024), at present there are 22 species of *Pteromalus* with a 4:4 mandibular formula worldwide, including eight species reported by Graham (1969) (*P.apum* (Retzius, 1783)) (as *P.venustus* Statz, 1938), *P.bifoveolatus* Förster, 1861, *P.procerus*, *P.proprius* Walker, 1874, *P.puparum* (Linnaeus, 1758) (type species of *Pteromalus*), *P.smaragdus* Graham, 1969, *P.squamifer* Thomson, 1878 and *P.vopiscus* Walker, 1839, six species described from Europe since 1969 (*P.bottnicus* Vikberg, 1979, *P.briani* Baur, 2015, *P.discors* Graham, 1992, *P.osmiae* Hedqvist, 1979, *P.paludicola* Bouček, 1972, and *P.sylveni* Hedqvist, 1979), three species from Kazakhstan (*P.melitaeae* Dzhanokmen, 1998, *P.maculatus* Dzhanokmen, 1998, and *P.transiliensis* Dzhanokmen, 1998), four species from China (*P.miyunensis* Yao & Yang, 2008, *P.orgyiae* Yang & Yao, 2015, *P.qinghaiensis* Liao, 1987, and *P.sanjiangyuanicus* Yang, 2020), and one species from North America (*P.quadridentatus* Gibson, 2024). *Pteromalussteppensis* Li & Hu, sp. nov. is different from the above 22 species and thus is newly described herein. The other new species, *P.xiaomoheensis* Yan & Li, sp. nov. is identified as belonging to the *P.albipennis* group of species and is separated from the existing species of that group based on Dzhanokmen (2001), Baur (2015), Klimmek and Baur (2018), and Maletti et al. (2021).

### ﻿Description two new species of *Pteromalus* from Xinjiang, China.

#### ﻿Key to the two new species and their similar species (Female)

**Table:** 

1	Both mandibles with four 4:4 (Fig. 1C)	**2**
–	Left mandible with three teeth and right mandible with four (Fig. 5D)	**3**
2	POL 1.10–1.14× OOL (Fig. 1F); distance between upper margin of antennal toruli and lower margin of median ocellus 1.00–1.42× as long as distance between lower margin of antennal toruli and lower margin of clypeus (Fig. 1B); antennae with scape reaching lower margin of anterior ocellus (Fig. 1F); combined length of pedicellus and flagellum shorter than breadth of head (0.73–0.84×) (Fig. 1B); medial area of propodeum smooth and shiny (Fig. 1G)	***Pteromalussteppensis* Li & Hu, sp. nov.**
–	POL 1.20–1.25× OOL; toruli about equidistant from the median ocellus and the lower margin of clypeus; antennae with scape reaching to level of vertex or slightly above it; combined length of pedicellus and flagellum almost equal to breadth of head; propodeum panels almost uniformly reticulate and not shiny	***P.procerus* Graham, 1969**
3	Body length 3.0–3.4 mm (Fig. 5A); lower margin of clypeus deeply incised medially, hence appearing almost bidentate (Fig. 5C); POL 1.50–1.86× OOL (Fig. 5G); the basal fovea of propodeum are relatively large (Fig. 5I); marginal vein length 1.09–1.13× longer than stigmal vein (Fig. 5J); gaster long-oval (Fig. 5L), 1.57–1.85× as long as broad, 1.06–1.30× as long as mesosoma	***Pteromalusxiaomoheensis* Yan & Li, sp. nov.**
–	Body length 2.7–3.1 mm (Fig. 6A); lower margin of clypeus moderately incised medially, hence without appearing bidentate (Fig. 6B); POL 1.40–1.50× OOL (Fig. 6C); the basal fovea of the propodeum are small (Fig. 6D); marginal vein 1.2–1.6× as long as the stigmal vein (Fig. 6E); gaster (Fig. 6F) short-oval, 1.2 to 1.6 times as long as broad, slightly shorter than to at most as long as the mesosoma	***P.tripolii* (Graham, 1969)**

##### ﻿ Pteromalus steppensis

Taxon classificationAnimaliaHymenopteraPteromalidae

Li & Hu sp. nov.

051876D1-F96E-542B-A6E4-8A4206AA3ADC

https://zoobank.org/B206A28A-E5AA-4ECB-9B1B-73037259EDC4

Fig. 1A–K

###### Type material.

***Holotype*.** • 1 ♀ (ICXU 20240801), Xinjiang, China, swept by Qin Li research group from *Ulmuspumila* L. (Ulmaceae) in People’s park of Changji City; 44°01'33.38"N, 87°18'42.67"E; 567 m; 24 May 2022and deposited in ICXU. ***Paratypes***: • 26 ♀ (ICXU 20240802–20240827), and 68 ♂ (ICXU 20240828–20240895), same collection site as holotype; China, Xinjiang, Urumqi, campus of Xinjiang University, Qin Li reared from the larva and pupa of *Orchestessteppensis* Korotyaev, 2016: • 5 ♀ (ICXU 20240896–20240900) 2 ♂ (ICXU 20240901–20240902), 30 May 2014; • 1 ♂ (ICXU 20240903), 31 May 2015; • 2 ♀ (ICXU 20240904–20240905), 2 Jun 2015; • 1 ♀ (ICXU 20240906), 3 Jun 2015; • 1 ♂ (ICXU 20240907), 4 Jun 2015; • 2 ♀ (ICXU 20240908–20240909), 7 Jun 2015; • 26 ♀ (ICXU 20240910–20240935), 8 Jun 2015; • 9 ♀ (ICXU 20240936–20240944), 9 Jun 2015; • 8 ♀ (ICXU 20240945–20240952), 12 Jun 2015; • 1 ♀ (ICXU 20240953), 15 Jun 2015; • 18 ♀ (ICXU 20240954–20240971) 14 ♂ (ICXU 20240972–20240985), 29.V.2016; • 5 ♀ (ICXU 20240986–20240990)13 ♂ (ICXU 20240991–20241003), 31 May 2016; • 2 ♀ (ICXU 20241004–20241005) 2 ♂ (ICXU 20241006–20241007), 3 Jun 2016; • 2♀ (ICXU 20241008–20241009), 8 Jun 2016; • 3 ♀ (ICXU 20241010–20241012) 1♂ (ICXU 20241013), 13 Jun 2016; • 2 ♀ (ICXU 20241014–20241015) 2 ♂ (ICXU 20241016–20241017), 23 Jun 2016.

###### Description.

**Figure 1.**
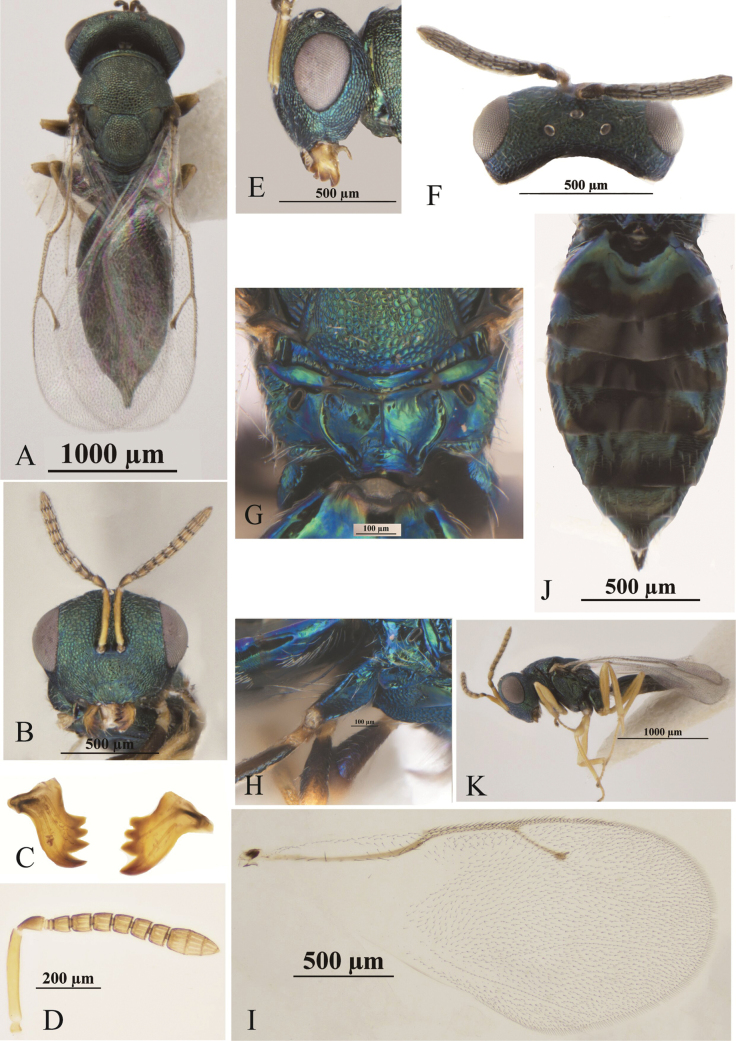
*P.steppensis* Li & Hu, sp. nov. **A–J** female **A** habitus, dorsal view **B** head, frontal view **C** mandible **D** antenna **E** head, lateral view **F** head, dorsal view **G** propodeum, dorsal view **H** hind coxa **I** forewing **J** metasoma, dorsal view **K** male, lateral view.

**Female.** Body (Fig. 1A) length 2.5–3.2 mm (*n* = 24). Head and mesosoma dark green, propodeum with bluish-green metallic reflections, metasoma dark green with shine, hind margin of Gt_1_, Gt_5_ and Gt_6_, mid-part of Gt_2_, Gt_3_ and Gt_4_ black. Antenna with yellowish-brown scape; dorsal side of pedicel and flagellum dark chocolate-brown; ventral side yellowish brown. Mandibles brown, with dark-brown teeth. Legs with all coxae dark, metallic green; femora dark-chocolate-brown, tibiae and tarsi yellowish brown except last segment black-brown. Wings hyaline, forewing with venation brown.

***Head*** in frontal view (Fig. 1B) 1.26–1.35× as broad as high, face with metallic reflections and regular raised-reticulation; clypeus (Fig. 1B) with longitudinal striations; lower margin moderately emarginate, without teeth; both mandibles (Fig. 1C) 4-toothed. Antennae (Fig. 1B, D) inserted at centre of face, higher than the lower margin of eyes; distance between upper margin of antennal toruli and lower margin of median ocellus 1.00–1.42× as long as distance between lower margin of antennal toruli and lower margin of clypeus; antennae with scape reaching lower margin of anterior ocellus, 7.25–7.63× as long as broad; pedicel 1.33–1.67× as long as broad in dorsal view, 1.71–1.98× as long as fu_1_; two anelli annular; funicular segments connected tightly to each other and each with one row of sensilla; fu_1_ and fu_2_ 1.00–1.20× as long as broad each, fu_3_ quadrate, fu_4_ and fu_5_ 0.79–0.87 as long as broad each, fu_6_ 0.64–0.69× as long as broad; clava 2.13–2.67× as long as broad; pedicle and flagellum combined 0.73–0.84× head width. Head in lateral view as in Fig. 1E; eye height 1.24–1.46× eye width; malar sulcus linear and complete; malar space 0.35–0.50× eye height. Head in dorsal view (Fig. 1F) about 2.13–2.45× as broad as long; POL 1.10–1.14× OOL (Fig. 1F).

***Mesosoma*** in dorsal view 0.84–0.87× head width; pronotum collar margined with carina, 0.71–0.85× as wide as mesoscutum, medially 1/9–1/7× as long as mesoscutum; mesoscutum 0.51–0.63× as long as broad; scutellum 0.90–1.00× as long as width, frenal line absent. Propodeum (Fig. 1G) 0.50–0.55× as long as scutellum, with medial area smooth; median carina and plica complete and sharp; nucha with fine transverse striations. Mesosoma in lateral view with prepectus smooth, 0.75–0.86× as long as tegula; entire thoracic pleura regularly reticulate, except upper mesepimeron smooth and shiny-metallic. Forewing (Fig. 1I) with apex exceeding apex of gaster, 2.01–2.24× as long as broad; upper surface of costal cell bare, lower surface of costal cell with 2–3 rows of setae interrupted medially; basal setal line incomplete and basal cell bare; speculum large and open posteriorly. Marginal vein length 0.96–1.00× postmarginal vein length and 1.43–1.68× stigmal vein length; postmarginal vein length 1.50–1.71× stigmal vein length. Metacoxa in dorsal view bare, with several long setaes (Fig. 1H), metafeumur 4.29–4.71× as long as broad.

***Gaster*** (Fig. 1J) long-oval, 1.98–2.38× as long as broad, 1.43–1.88× as long as mesosoma, 1.13–1.21× as long as head plus mesosoma; Gt1 0.20–0.25× as long as gaster and sides of Gt1 with some sparse long setaes; Gt7 0.62–0.73× as long as broad; ovipositor sheaths slightly exserted; hypopygium extending about 0.38–0.45× the length of gaster.

**Male** (Fig. 1K). Body length: 3.0–3.2 mm. Body color: head, mesosoma, and metasoma the same color as the female; antenna yellowish brown; legs yellow, except all coxae dark green with metallic reflections and apical tarsi black-brown. Wings hyaline, forewing with venation brown.

***Head*** in frontal view 1.25–1.35× as broad as high; distance between upper margin of antennal toruli and lower margin of median ocellus 0.92–1.16× as long as distance between lower margin of antennal toruli and lower margin of clypeus; pedice and flagellum combined 0.89–0.96× head width. Head in dorsal view 2.08–2.29× as broad as long; POL 1.19–1.29× OOL.

***Mesosoma*** in dorsal view 0.79–0.81× as head width; pronotum 0.81–0.85× as wide as mesoscutum; mesoscutum 0.66–0.67× as long as broad. Propodeum 0.49–0.54× as long as scutellum. Forewing with marginal vein length 1.03–1.19× postmarginal vein length and 1.48–1.88× stigmal vein length; postmarginal vein length 1.44–1.60× stigmal vein length.

***Gaster*** long-oval, 1.89–2.18× as long as broad, 0.77–0.84× as long as head plus mesosoma; Gt1 about 0.35–0.39× as long as gaster.

###### Etymology.

The species is named after its host species *Orchestessteppensis* Korotyaev, 2016 (Coloptera, Curculionidae) (used as a noun in apposition).

###### Biology.

This species was reared as a primary, solitary ectoparasitoid of the larval and pupal stage of *O.steppensis* Korotyaev, 2016. Figures of the development and its damage on the host are provided in Figs 3, 4.

###### Distribution.

Changji and Urumqi, Xinjiang, China.

###### Comments.

This species is similar to *P.procerus*. In females of that species: both mandibles with four teeth; anterior margin of clypeus shallowly emarginate, hardly impressed in the middle; clypeus strigose, the striae hardly extending on to the face and genae; pronotal collar distinctly less wide than the mesoscutum, shorter medially than at the sides, medially from slightly more than 1/8–1/7 as long as the mesoscutum, finely reticulate with a narrow shiny strip along its hind edge, slightly to distinctly margined anteriorly; propodeum somewhat more than half as long as the mesoscutellum (Graham 1969: fig. 389); gaster lanceolate or sublanceolate, usually as long as or slightly longer than the head plus thorax, occasionally slightly shorter, 1.8–2.3 times as long as broad. However, the new species can be distinguished from *P.procerus* by the following: POL 1.10–1.14× OOL; distance between upper margin of antennal toruli and lower margin of median ocellus 1.00–1.42× as long as distance between lower margin of antennal toruli and lower margin of clypeus; antennae with scape reaching lower margin of anterior ocellus; combined length of pedicellus and flagellum shorter than breadth of head (0.73–0.84×); medial area of propodeum smooth and shiny. In *P.procerus*: POL 1.20–1.25× OOL; toruli about equidistant from the median ocellus and the lower margin of clypeus; antennae with scape reaching to level of vertex or slightly above it; combined length of pedicellus and flagellum almost equal to breadth of head; propodeum panels almost uniformly reticulate and not shiny.

**Figure 2.**
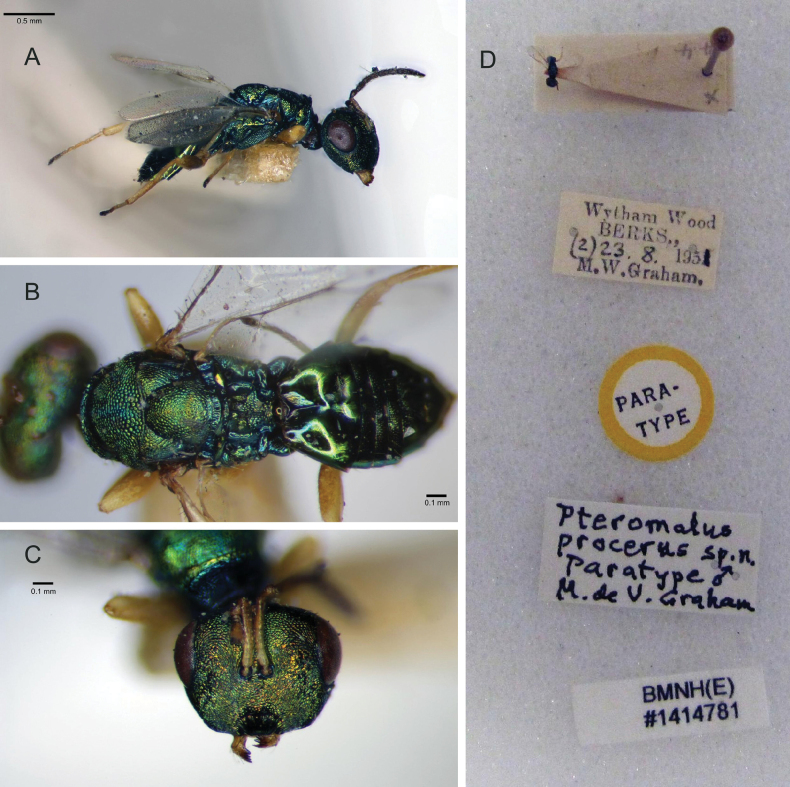
*P.procerus* Graham, 1969 **A–C** male **A** body, lateral view **B** mesosoma and metasoma, dorsal view **C** head, frontal view **D** paratype information.

In males of *P.steppensis* Li & Hu, sp. nov. (Fig. 1K): antenna yellowish brown; legs yellow, except all coxae dark green with metallic reflections, and apical tarsi black-brown; propodeum smooth and shiny, 0.49–0.54× as long as scutellum. In contrast, in *P.procerus* (Fig. 2A–D): antenna dark brown to black; pale parts of the legs yellowish, hind femora lightly infuscate at the base only and the fore and mid femora yellowish; propodeum reticulated, about 2/3× as long as scutellum.

**Figure 3.**
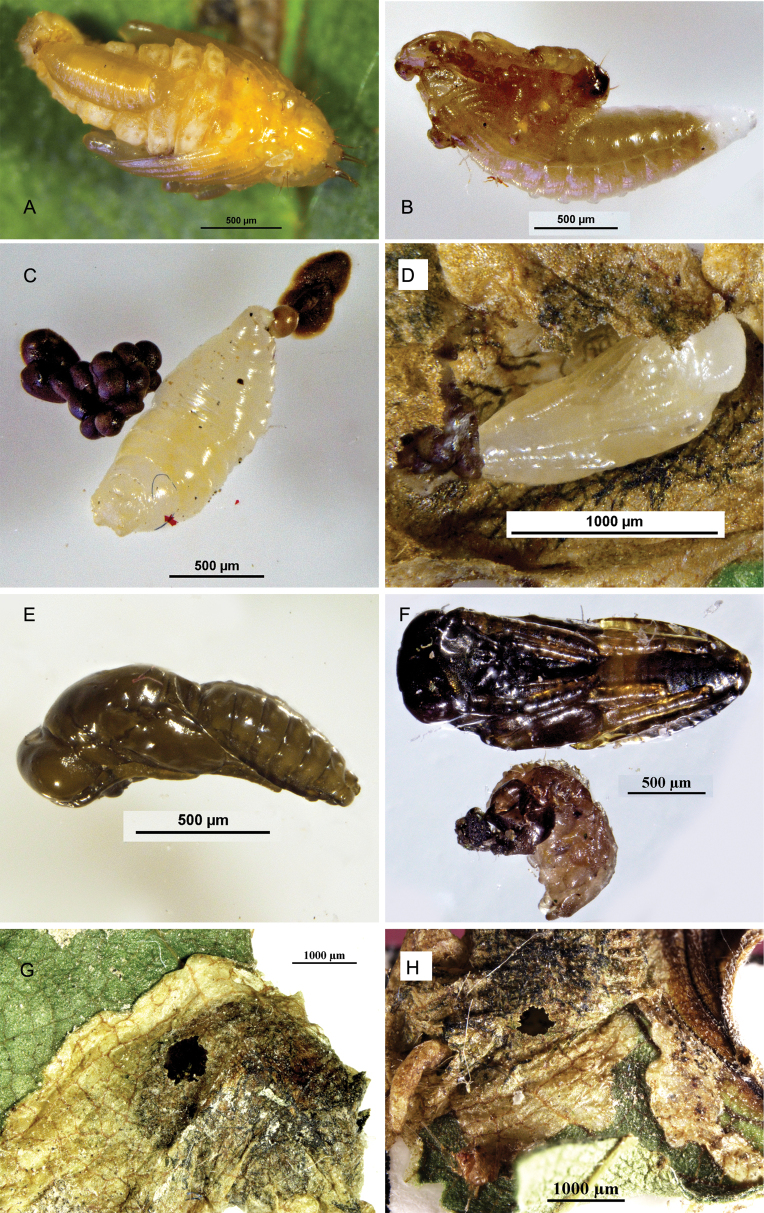
Development stages of *P.steppensis* Li & Hu, sp. nov. and their emergence holes, **A–H**. **A, B** the parasitoid larva feeding on the third larva of the host **C** prepupa **D–F** pupa **G, H** emergence holes.

**Figure 4.**
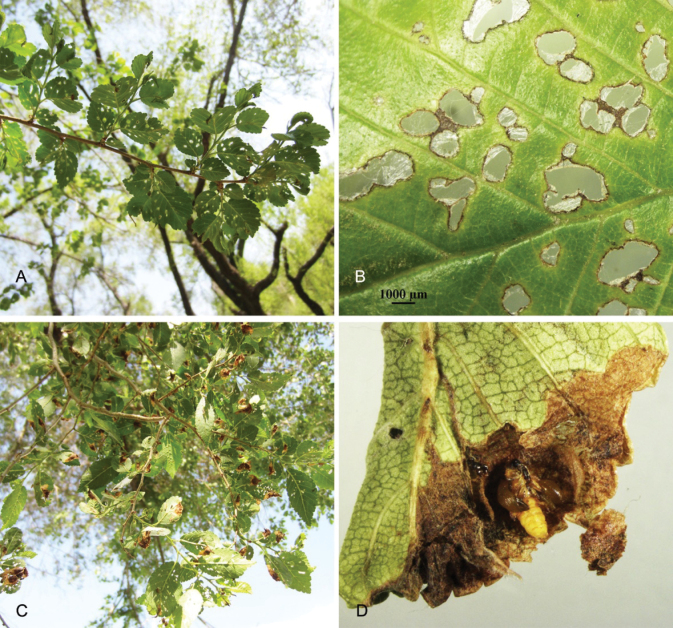
Damage on *Ulmuspumila* leaves made by the adults as well as the larvae of *O.steppensis* Korotyaev, 2016 **A–D**. **A, B** holes on *U.pumila* leaves made by the adults **C, D***U.pumila* leaves.

##### ﻿ Pteromalus xiaomoheensis

Taxon classificationAnimaliaHymenopteraPteromalidae

Yan & Li sp. nov.

698E56FC-4F1B-55CD-B4BA-0240534C41DB

https://zoobank.org/A52C92F0-6305-431B-B59C-AC9BAF665E4C

Fig. 5A–L

###### Type material.

***Holotype*.** • 1 ♀ (ICXU 20246442), Xinjiang, China, Gongliu County of Yili Prefecture; 43°10'52.04"N, 82°44'9.49"E; 1377 m; 10 Jul 2021, coll. Liqin research group. ***Paratypes*.** • 8 ♀ (ICXU 20246443–20246450), same collection information as holotype; • 2 ♀ (ICXU 20246451–20246452), Qinghe County of Altay Prefecture; 46°26'5.83"N, 90°2'45.15"E; 600 m; 9 Jul 2020, coll. Liqin research group; • 1 ♀ (ICXU 20246453), Altai City of Altay Prefecture; 47°39'26.97"N, 88°17'20.22"E; 624 m; 24 Jun 2021, coll. Liqin research group; • 1 ♀ (ICXU 20246454), Fuyun County of Altay Prefecture; 47°1'7.69"N, 89°45'24.35"E; 830 m; 11 Jul 2020, coll. Liqin research group; • 6 ♀ (ICXU 20246455–20246460), Zhaosu County of Yili Prefecture; 43°9'10.43"N, 81°26'38.83"E; 1556 m; 9 Jul 2021, coll. Liqin research group; • 1 ♀ (ICXU 20246461), Xinyuan County of Yili Prefecture; 43°22'38"N, 83°36'18"E; 1279 m; 28 Jul 2018, Coll. Hongying Hu research group; • 1 ♀ (ICXU 20246462), Tekes County of Yili Prefecture; 43°13'19.12"N, 81°48'31.80"E; 1200 m; 8 Jul 2021, coll. Liqin research group; • 33 ♀, Tekes County of Yili Prefecture; 43°9'19.98"N, 88°47'23.76"E; 1184 m; 9 Jul 2021, coll. Liqin research group; • 1 ♀ (ICXU 20246463), Gongliu County of Yili Prefecture; 43°20'22.85"N, 82°31'4.19"E; 881 m; 10 Jul 2021, coll. Liqin research group; • 1 ♀ (ICXU 20246464), Urumqi County of Urumqi; 43°27'25.02"N, 87°22'37.20"E; 1757 m; 5 Jul 2022, Coll. Hongying research Hu.

###### Description.

**Figure 5.**
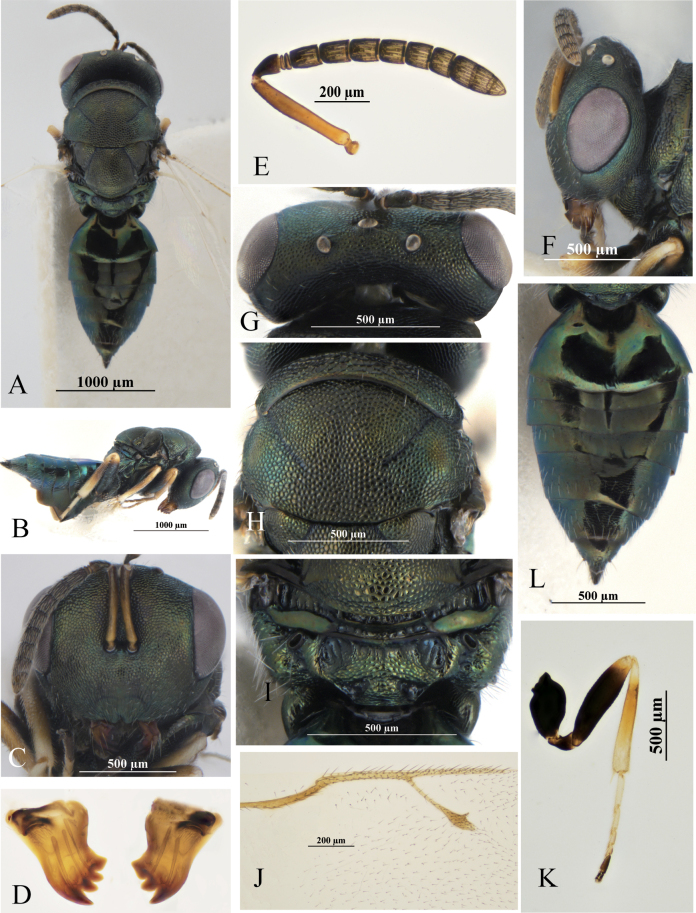
*P.xiaomoheensis* Yan & Li, sp. nov. holotype **A–L** female **A** habitus, dorsal view **B** body, lateral view **C** head, frontal view **D** mandible **E** antenna **F** head, lateral view **G** head, dorsal view **H** mesoscutem, dorsal view **I** propodeum, dorsal view **J** forewing **K** hind leg **L** metasoma, dorsal view.

**Female.** Body (Fig. 5A, B) length 3.0–3.4 mm (*n* = 5). Head and mesosoma dark metallic-green, metasoma dark green with reflections. Antenna with yellowish-brown scape; pedicel and flagellum dark-chocolate-brown. Legs (Fig. 5K) with all coxae dark metallic-green, femora dark green, except for apical part brown, tibiae yellowish brown to brown and tarsi pale yellow, except last segment brown. Wings hyaline; forewing with venation brown.

***Head*** in frontal view (Fig. 5C) 1.19–1.28× as broad as high, face with metallic reflections and regular raised-reticulations; clypeus (Fig. 5C) with longitudinal striations, with lower margin deeply incised medially, hence appearing almost bidentate; left mandible 3-toothed and right mandible 4-toothed (Fig. 5D). Antennae (Fig. 5C) inserted at centre of face, higher than the lower margin of eyes; distance between upper margin of antennal toruli and lower margin of median ocellus 0.91–1.11× as long as distance between lower margin of antennal toruli and lower margin of clypeus; antennae with scape reaching lower margin of anterior ocellus; pedicel 1.55–1.59× as long as broad in dorsal view; two anelli annular; funicular segments (Fig. 5E, G) connected tightly to each other and each with two rows of sensilla; fu_1_ to fu_4_ each about 1.10–1.30× as long as broad each; fu_5_ 0.95–1.12× as long as broad; fu_6_ 0.73–0.77× as long as broad; clava 2.52–2.68× as long as broad; pedicle and flagellum combined 0.75–0.90× head width. Head in lateral view (Fig. 5F), eye height 1.42–1.70× as eye width; malar sulcus linear and complete, and malar space 0.46–0.48× eye height. Head in dorsal view (Fig. 5G), 2.19–2.44× as broad as long; POL 1.50–1.86× OOL.

***Mesosoma*** in dorsal view (Fig. 5A) 0.86–0.96× as head width; pronotum collar (Fig. 5H) margined with carina, 0.88–0.91× as wide as mesoscutum, medially one quarter as long as mesoscutum; mesoscutum 0.62–0.72× as long as broad; scutellum 0.95–1.00× as long as width, frenal line absent. Propodeum (Fig. 5I) 0.35–0.47× as long as scutellum, medial area reticulated; median carina and plica complete and sharp; nucha large and reticulate. Mesosoma in lateral view with prepectus smooth, 0.62–0.80× as long as tegula; entire thoracic pleura regularly reticulate, except the upper mesepimeron smooth and shiny metallic. Forewing (Fig. 5J) apex exceeding apex of gaster, 2.05–2.14× as long as broad; lower surface of costal cell with one row of hairs interrupted medially; basal setal line and basal cell bare; speculum large and open posteriorly. Marginal vein length 0.96–1.04× postmarginal vein length and 1.09–1.13× longer than stigmal vein length; postmarginal vein 1.10–1.14× as long as stigmal vein. Metacoxa in dorsal view bare, metafumur (Fig. 5K) 3.87–4.16× as long as broad.

***Gaster*** long-oval (Fig. 5L), 1.57–1.85× as long as broad, 1.06–1.30× as long as mesosoma, 0.81–0.97× as long as head plus mesosoma; Gt_1_ about 0.31–0.35× as long as gaster; Gt_7_ 0.63–1.14× as long as broad; ovipositor sheaths exserted, length of extend part 0.5–1.25× as long as Gt_7_ length; hypopygium extending about 0.51–0.67× the length of gaster.

**Male.** Unknown.

###### Etymology.

The species is named after the collection locality of its holotype.

###### Biology.

Unknown.

###### Distribution.

Xinjiang, China: Yili Prefecture (Gongliu County; Zhaosu County; Xinyuan County; Tekes County; Gongliu County), Altay Prefecture (Qinghe County; Altai City) and Urumqi.

###### Comments.

**Figure 6.**
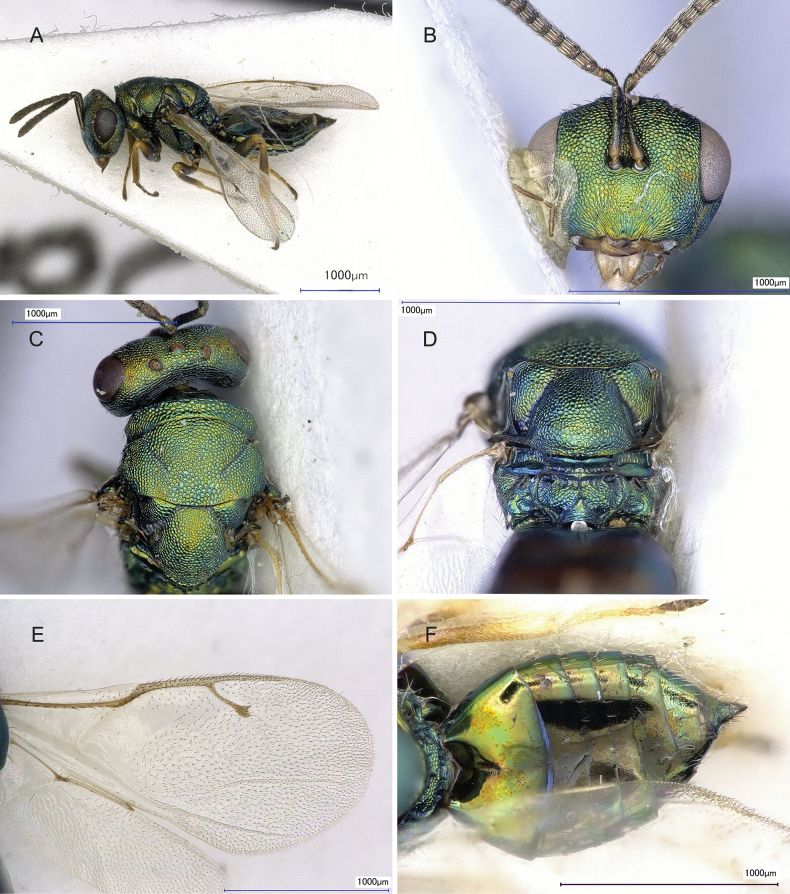
*P.tripolii* (Graham, 1969) **A–F** female **A** body, lateral view **B** head, frontal view **C** head and mesosoma, dorsal view **D** propodeum, dorsal view **E** forewing **F** gaster, dorsal view.

This species is similar to *P.tripolii* (Fig. 6A–F); both species belong to the *albipennis* species group, both have with left mandible with three teeth and right mandible with four; head and thorax brightly metallic, green to blue, brassy, or coppery; antenna with sensilla usually numerous and in two irregular rows on at least the proximal segments of the funicle; fu_1_ longer than broad and fu_6_ quadrate or transverse; combined length of pedicellus and flagellum distinctly less than the breadth of the head; propodeum medially a little less than half as long as the scutellum (Graham 1969: fig. 377); gaster ovate, basal tergite occupying 1/3–2/5 of the total length. However the new species is distinguished from *P.tripolii* by the following characters: body color dark blue; body length 3.0–3.4 mm; gaster long-oval (Fig. 5L), 1.57–1.85× as long as broad, 1.06–1.30× as long as mesosoma. In *P.tripolii*: body color usually bright green to blue (Fig. 6A); body length 2.7–3.1 mm (Fig. 6A); gaster (Fig. 6F) short-oval, about as long as, or slightly shorter than, the mesosoma, about as broad as the latter, 1.2 to 1.6 times as long as broad, acute but not acuminate apically (Fig. 6A).

## ﻿Discussion

This study adds significant knowledge on the faunal composition and distribution of *Pteromalus* species in China. With the description of two new species from western China (Eastern Palaearctic Region), the genus now includes 496 valid species worldwide, with 21 of these in China and six species in Xinjiang. As reported by Gibson et al. (2024: p202) “Described species of *Pteromalus* with a 4:4 mandibular formula appear to be far fewer in number than those with a 3:4 formula. For example, of the 67 species Graham (1969) treated from northwestern Europe, eight named species were reported under *Pteromalus* and 59 named species under *Habrocytus* (12%)’’. We had similar results, with only nine Chinese species of *Pteromalus* having four teeth on both mandibles, and 12 species with three teeth on the left mandible and four teeth on the right mandible.

*Pteromalussteppensis* Li & Hu, sp. nov. is described from both sexes and was reared as a primary, solitary ectoparasitoid of larval and pupal stages of *Orchestes. Steppensis*. Detailed information on its life history and phenology of its host in Xinjiang, images of its larval and pupal stages, as well as of its host’s life habits can be found in the publication by Li et al. (2018). Almost 10 years have passed since the first author (Qin Li) reared 558 (340 ♀, 218 ♂) specimens of this new species from its host *O.steppensis*. Li et al. (2018) reported this new species as *Pteromalus* sp. 2. Guohua Yan studied the integrated taxonomy of *Pteromalus* from Xinjiang based on three methods, including morphological taxonomy, comparative morphology, and DNA Barcoding (28S rDNA and ITS2 genes) (Li et al. 2018). She collected 95 specimens (27 ♀, 68 ♂) of this new species by sweep netting on *Ulmuspumila* L. (Ulmaceae) heavily infested by the pest, *O.steppensis* in People’s park of Changji City, Urumqi, Xinjiang, China. She compared her specimens with the specimens reared by Qin Li from the elm pest, *O.steppensis* and based on its morphological characters, and confirmed they are the same species, *P.steppensis* Li & Hu, sp. nov. These results indicate that many more investigations are necessary to have a complete overview of the *Pteromalus* fauna from China.

## Supplementary Material

XML Treatment for Pteromalus steppensis

XML Treatment for Pteromalus xiaomoheensis

## ﻿Additional information

### Author ORCIDs

Qin Li https://orcid.org/0000-0002-1780-7332

Hong-ying Hu https://orcid.org/0000-0002-2295-1072

### Data availability

All of the data that support the findings of this study are available in the main text.
